# Development of the Japanese version of the three-item loneliness scale

**DOI:** 10.1186/s40359-019-0285-0

**Published:** 2019-04-05

**Authors:** Tasuku Igarashi

**Affiliations:** Graduate School of Education and Human Development, Nagoya University, Furo-cho, Chikusa-ku, Nagoya, Aichi 464-8601 Japan

**Keywords:** Loneliness, Short-form scale, Japanese version, Item response theory

## Abstract

**Background:**

Loneliness is a major risk factor for mental and physical health worldwide. The Three-Item Loneliness Scale (TIL Scale; Hughes et al., 2004) has been widely applied to measure loneliness in a simplified format, but no validated Japanese version has been developed. This study adapted the TIL Scale into Japanese and tested its reliability and validity.

**Methods:**

The original English version of the TIL Scale was translated into Japanese, and the expressions of the Japanese version were confirmed by a back translation procedure. The translated scale was then administered to Japanese respondents recruited from an online research panel (*N* = 1020) and an online crowdsourcing service (*N* = 500). To analyze the data containing polytomous responses to the items in the scale, this study used categorical Confirmatory Factor Analysis and the Generalized Partial Credit Model based on the Item Response Theory. To evaluate the psychometric properties of the scale, this study examined factorial validity, reliability, information curves, and the associations of the scale score with demographic variables (age, gender, marital status, and living arrangements), the scores of the Revised UCLA Loneliness Scale (R-UCLA), the Big Five scale of personality traits, and the sizes of personal networks.

**Results:**

The translated TIL Scale showed essential unidimensionality and characteristics to differentiate among respondents at different levels of loneliness. The scale score was related positively with the scores of R-UCLA and neuroticism and negatively with the scores of extraversion, conscientiousness, openness, agreeableness, and the sizes of overall and support networks. Those who were unmarried and alone recorded a higher score on the scale than those who were married and living with someone. Age showed a negative correlation with the scale score only in Sample 1, in which the equal allocation procedure was introduced for the age stratum.

**Conclusions:**

The results indicate that the Japanese version of the TIL Scale demonstrates adequate reliability and validity for the assessment of loneliness.

**Electronic supplementary material:**

The online version of this article (10.1186/s40359-019-0285-0) contains supplementary material, which is available to authorized users.

## Background

Loneliness has become a pervasive social phenomenon around the world. Conceptualized as a subjective negative feeling arising from the perception of a discrepancy between one’s actual and ideal states of interpersonal relationships [1], loneliness is known not only to harm physical and mental health [2], but also to relate to poor structural social relationships, including single marital status and living alone, both of which could increase mortality risks [3]. Although social isolation and loneliness are distinct concepts, deficits of social supports with close others increase loneliness [4]. Reliance on online social relationships could also increase loneliness if people use them to compensate for their insufficient face-to-face relationships [5]. In 2018, the United Kingdom appointed a minister to address the problem of loneliness as a national policy [6]. Understanding the nature of loneliness is an issue that calls for urgent attention to improve the well-being of individuals and the vitality of societies.

Previous studies have mainly used the 20-item Revised UCLA Loneliness Scale (R-UCLA) to assess loneliness [7]. Although R-UCLA is a well-established and standard measure of loneliness and has been utilized since it was developed in 1980, there is a growing concern on a short-form psychological scale to reduce questionnaire length to increase response rate in particular circumstances, such as clinical practice, longitudinal surveys, and pre-screenings [8, 9]. The Three-Item Loneliness Scale (TIL) was established based on R-UCLA to mitigate the response burden and was validated by the associations with several indicators, such as R-UCLA scores, depressive symptoms, marital status, and living arrangements [10]. A recent study found associations between scores on the TIL Scale and the Big Five personality traits, in which neuroticism is found to be a major determinant of loneliness based on genetic analysis [11]. Since its first development for a telephone survey to old adults in the U.S., the scale has been applied in various settings, including undergraduate studies [12], large-scale longitudinal panel studies [11], and online surveys [13].

This study aims to develop a Japanese version of the TIL Scale. As in the West, loneliness has become a significant social issue in Japan, where the number of single-occupancy households has been increasing among old adults, and solitary deaths are not uncommon, even among the youth and the middle-aged [14]. Systematic comparisons of scientific evidence in loneliness across different cultures are important to fully understand the nature of the psychological concept. Although the Japanese version of R-UCLA has been utilized since its development in 1983 [15], there is no doubt about the need for a short-form loneliness scale in Japanese for both practical and academic purposes.

Whereas loneliness has been regarded as a major problem among older adults [16], recent research has also shown high levels of loneliness among youth who typically face several developmental tasks such as socialization, friendship reconstruction, and emotion regulation [17]. In terms of gender differences in loneliness, past research has yielded mixed results. Some studies reported higher levels of loneliness among females than males [4], while others reported no gender difference [18, 19]. Also, living alone is found to be a significant predictor of loneliness [20]. This study translated the original TIL scale into Japanese and evaluated its psychometric properties in consideration with age, gender, and living arrangements as possible determinants of loneliness.

## Method

### Respondents

Data were collected in Japan from two different samples, for a form of cross-validation in which one sample is used for model construction and the other sample is used for confirmation and validation of the model. Sample 1 consisted of 1020 respondents (510 males and 510 females aged 13 to 80; mean age =36.1 (*SD* = 14.5)) recruited through an online research panel. The panel was provided by Goo Research, a major online research service in Japan managed by NTTCom Online Marketing Solutions Corporation. The service provides an online research panel of over three million Japanese people and is able to contact potential participants via email if researchers request the collection of survey data from specific strata. Participants in Sample 1 agreed to join the study after receiving an email notification about the survey from Goo Research (https://research.nttcoms.com/), which described the study, and subsequently deciding to opt-in. Informed consent was obtained online prior to taking part in the study. For the participants under the age of 18, Goo Research confirms that they obtained permission from a person with parental authority to register for the service and take part in any surveys on the service. Sample 2 consisted of 500 respondents (195 males and 305 females aged 18 to 73; mean age = 37.9 (*SD* = 9.9)) recruited through an online service. This service was provided by Lancers (https://www.lancers.jp/), a major crowdsourcing service in Japan managed by Lancers, Inc. The online service coordinates requests from clients with crowdsourcing workers. The current research was posted by the author as a psychology research project in the "research" category on the Lancers information board. The participants read the descriptions of the study and agreed to take part in it by opting into the study themselves.

To examine the impacts of these demographic variables on loneliness, the study used an equal size allocation procedure for age, gender, and living arrangements strata in Sample 1, which sampled approximately equal numbers of respondents from the teens (*N* = 204), twenties (*N* = 204), thirties (*N* = 204), forties (*N* = 205), fifties and above (*N* = 203), males and females (*N* = 510, respectively), and those living alone and living with someone (*N* = 510, respectively).

### Measurement instruments

#### Japanese version of the TIL scale

To develop the Japanese version of the TIL Scale, the study used a back translation procedure. With permission from a member of the research team who developed the original TIL Scale, the author of this article (TI), a native Japanese speaker who had spent 2 years as a postdoctoral researcher in an English-speaking country, initially translated the original instruction and items into Japanese. The original English version is intended to be used in phone interviews, but the translated version is intended to be used in pen-and-pencil/online surveys. Therefore, expressions in the translated version were altered slightly from the original to fit the purpose (e.g., interrogative sentences were changed to active sentences). Then, an international graduate student, a native speaker of English who obtained a master’s degree in psychology in Japan, independently conducted a back translation from Japanese to English with no knowledge of the concept of the scale. Discrepancies between the expressions in the two initial and back translations were discussed in detail and carefully resolved until the initial and back translations were closely matched. The final version of the Japanese version of the TIL Scale is shown in Additional file 1: Table S1. Each item is rated on a 3-point Likert scale (1: Hardly ever, 2: Some of the time, and 3: Often), and the scale score ranged from 3 to 9.

#### Other measures for scale validation

##### The revised UCLA loneliness scale (R-UCLA) (sample 2)

The R-UCLA [7] is the original version of the TIL Scale and one of the most widely used scales in Japan [15] to measure subjective feelings of chronic loneliness. The scale consists of 20 items, and respondents rated the degree of loneliness on a 4-point Likert scale (1: Never to 4: Often; the scale score ranged from 20 to 80).

##### The big five scale of personality traits (sample 2)

The Big Five scale of personality traits [21] is commonly used in Japan to assess personality traits based on the five-factor model of personality. This study used a short version of the scale [22], which consists of 29 adjectives rated on a 7-point Likert scale (1: Strongly disagree to 7: Strongly agree) to measure extraversion (five items; the scale score ranged from 5 to 35), neuroticism (five items; ranged from 5 to 35), conscientiousness (seven items; ranged from 7 to 49), openness (six items; ranged from 6 to 42), and agreeableness (six items; ranged from 6 to 42).

##### Personal networks (sample 1)

This study assessed the sizes of four different personal networks^1^: overall networks (number of people listed in respondent’s cellphone/smartphone address book), support networks (number of family members/friends/acquaintances with whom personal matters can be discussed), online networks (number of acquaintances met and communicated with only online), and new-acquaintance networks (number of acquaintances newly met in the last month). Each network was measured by one item.

### Analytic procedure

Analyses on the translated TIL Scale were conducted in the following way: First, I analyzed the data obtained from Sample 1 to check the descriptive statistics of each item in the scale and test the unidimensionality and factor structure of the scale. Second, I analyzed the data obtained from Sample 1 to examine the characteristics of the items in the scale based on the Item Response Theory. Finally, I analyzed the data obtained from Sample 2 and part of the data obtained from Sample 1 to confirm the validity of the scale by checking its associations with theoretically-related variables.

## Results

### Descriptive statistics

Table 1 shows descriptive statistics of the items in the translated TIL Scale (Sample 1). Most respondents chose the middle point of the ratings (2), and the distribution of the item scores was not strongly skewed (all skewness < 1). All items showed moderate to strong positive correlations with each other.

**Table 1:** Descriptive statistics and results of confirmatory factor analysis of the translated TIL Scale (Sample 1)

	*M*	*SD*	*Med.*	*Min.*	*Max.*	Skewness	Kurtosis	Correlations			Factor 1
								1	2	3	
1. I feel that I lack companionship.	1.96	0.68	2	1	3	0.04	-0.81	-			.72
2. I feel left out.	1.63	0.65	2	1	3	0.54	-0.69	.63	-		.88
3. I feel isolated from others.	1.82	0.69	2	1	3	0.25	-0.90	.69	.83	-	.95
Three-Item Loneliness Scale (Total)	5.42	1.72	6	3	9	0.36	-0.52	.81	.86	.89	

*N* = 1020; Correlations among the items are polychoric; Correlations of the items with the total score are Pearson's *r*

### Factor structure

To confirm unidimensionality of the translated TIL Scale, I conducted a categorical confirmatory factor analysis using the weighted least squares with mean and variance adjustment method based on polychoric correlations of the items (Sample 1). The model included a single latent variable having three items of the translated TIL Scale as observed variables (i.e. a saturated model). All items had factor loadings of more than .70 for the first factor (see Table 1). Eigenvalues (calculated based on polychoric correlations) were 2.44, 0.40, and 0.16, indicating that the first factor explains 81% of the total variance. Cronbach’s alpha coefficient of the scale was .81. These results clearly revealed the unidimensionality of the scale, which satisfies the assumption of IRT.

### IRT analysis

The translated TIL Scale was analyzed using Muraki’s Generalized Partial Credited Model (GPCM) [23]. PARSCALE 4.1 [24] was used to estimate parameters. GPCM can be applied to polytomous items with multiple-ordered response categories and specify slope, location, and category parameters for each item.^2^ The slope (discrimination) parameter indicates the degree to which an item can discriminate a latent trait (*θ*; e.g., loneliness) among respondents. The mean of *θ* is set as zero and the standard deviation as one. The location (difficulty) parameter indicates the degree to which an item (e.g., “I feel left out.”) involves a difficulty for respondents to give higher ratings (e.g., 3: Often) regardless of the level of a latent trait (e.g., loneliness). The category parameter is related to points of intersection between Item Response Category Characteristics Curves (IRCCC) for each item depicted based on the slope and location parameters. IRCCCs represent the probability that those with a particular level of a latent trait (e.g., high in loneliness) choose a particular response category (e.g., 3: Often) for an item (e.g., “I feel left out.”). An Item Information Curve (IIC) is obtained from the summation of the IRCCCs at each value of *θ* for an item and indicates how accurate (e.g., rich in information) the item (e.g., “I feel left out.”) is to measure a certain level of a latent trait (e.g., high in loneliness). A Test Information Curve (TIC) is depicted as the summation of the IICs at each value of *θ* for all items in the scale and provides information about a discriminative power of the whole scale (e.g., TIL) for a latent trait (e.g., loneliness).

**Fig. 1:**
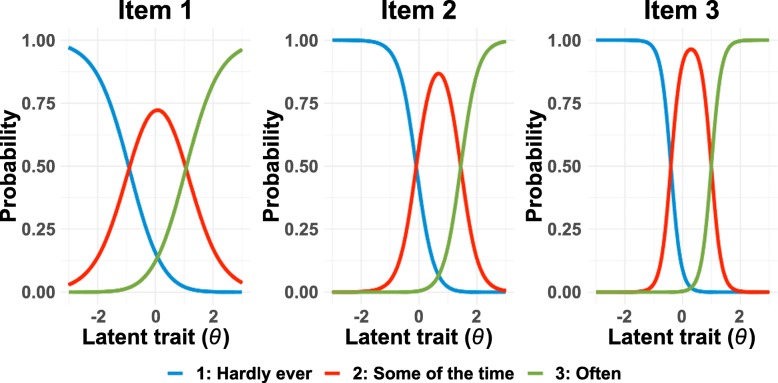
IRCCC for each item in the translated TIL Scale (Sample 1)

Table 2 shows estimates of slope, location, and category parameters for each item of translated TIL Scale (Sample 1), and Fig. 1 shows the IRCCC of each item. The slope parameters of Items 2 and 3 were relatively large, indicating a discriminative characteristic of the items for those high and low in loneliness. The location parameter of Item 2 was positive and relatively large, indicating a difficulty for respondents to rate themselves as “left out.” Meanwhile, both the slope and location parameters of Item 1 were relatively low, showing a poorer discriminative characteristic of the item than the other items for those high and low in loneliness. In other words, those low in loneliness did not have difficulty in rating themselves as having the characteristic described in the item (i.e., lacking companionship).

**Table 2:** IRT Parameters for each item of the translated TIL Scale estimated in GPCM (Sample 1)

	Slope		Location		Category 1		Category 2	
1. I feel that I lack companionship.	0.983	(0.055)	0.077	(0.049)	0.986	(0.053)	-0.986	(0.053)
2. I feel left out.	1.967	(0.181)	0.676	(0.041)	0.769	(0.029)	-0.769	(0.029)
3. I feel isolated from others.	3.354	(0.529)	0.294	(0.038)	0.701	(0.022)	-0.701	(0.022)

Values in parentheses indicate standard errors of the estimates

**Fig. 2:**
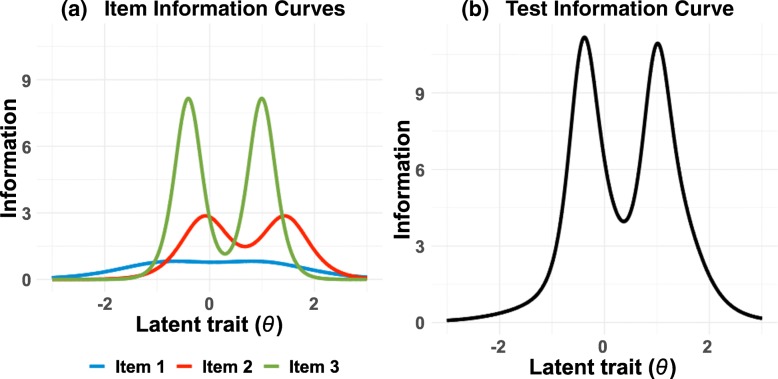
(**a**) IIC and (**b**) TIC of the translated TIL Scale (Sample 1)

Figure 2 shows the IIC of each item and the TIC of the translated TIL Scale. The IICs of Items 2 and 3 and the TIC of the scale were bimodal with two peaks. The TIC produced maximal information at about *θ*s *=* − 0.4 and 1.0, corresponding to 4 to 5 and 7 to 8 on the 7-point rating (range: 3–9) of TIL, respectively.

### Local independence

Local independence is a basic assumption of IRT that item responses should be conditionally independent of each other and related only via a latent trait. To assess local independence of the items in the TIL scale, *Q*_3_ statistics were calculated. Item 3 showed slight tendencies of local dependence (> .20) with Items 1 and 2 (*Q*_3_ = .22 and .26, respectively).

### Test for convergent validity

Table 3 represents the correlation coefficients of the score on the translated TIL Scale (i.e., the loneliness score) with relevant variables in Samples 1 and 2. The correlation of the loneliness score with the original R-UCLA score was strong and positive. No gender difference was found in the loneliness score in both samples. In terms of age, younger respondents reported a higher level of loneliness in Sample 1, but this pattern was not observed in Sample 2. Supplementary regression analysis on the loneliness score by using dummy variables of age (baseline = teens) revealed that the teens showed higher loneliness scores than those in their thirties and above, while no difference was found between the teens and twenties [see Additional file 1: Table S2]. Unmarried respondents in Sample 1 reported a higher level of loneliness than married respondents, and those who lived alone in Sample 1 reported a higher level of loneliness than those who lived with someone.

**Table 3:** Correlation coefficients of the translated TIL Scale with relevant variables (Samples 1 and 2)

			Sample 1 (*N* = 1020)		Sample 2 (*N* = 500)	
	*M*	(*SD*)	Pearson's *r*	95%CI	Pearson's *r*	95%CI
Three-Item Loneliness Scale (TIL Scale; Sample 2)	5.72	(1.81)				
Revised UCLA Loneliness Scale (R-UCLA)	47.8	(10.3)			.771**	[.730, .800]
Demographic variables						
Gender (1 = Female)			-.036	[-.100, .020]	-.070	[-.160, .020]
Age			-.172**	[-.230, -.110]	-.040	[-.130, .050]
Marital status (1 = Married)			-.194**	[-.250, -.130]		
Living arrangements (1 = Living with someone)			-.162**	[-.220, -.100]		
Personal network size						
Overall network size	95.0	(106.4)	-.167**	[-.230, -.110]		
Support network size	4.98	(5.08)	-.231**	[-.290, -.170]		
Online network size	5.58	(27.2)	.029	[-.030, .090]		
New-acquaintance network size	4.33	(14.7)	-.026	[-.090, .040]		
Big Five scale of personality traits						
Extraversion	19.3	(6.22)			-.530**	[-.590, -.460]
Neuroticism	25.0	(5.91)			.426**	[.350, .500]
Conscientiousness	28.2	(7.24)			-.174**	[-.260, -.090]
Openness	25.0	(6.20)			-.253**	[-.330, -.170]
Agreeableness	25.3	(5.55)			-.373**	[-.450, -.300]

**p < .01; Means and standard deviations (*SD*) are presented for network and psychological measures

Regarding personal networks, the sizes of overall and support networks were negatively correlated with the loneliness score in Sample 1, whereas those with online and new-acquaintance networks were not. All dimensions of Big Five personality traits showed significant associations with the loneliness score in Sample 2, with which neuroticism was positively correlated; and extraversion, conscientiousness, openness, and agreeableness were negatively correlated, respectively.

## Discussion

This study developed the Japanese version of the TIL Scale. The factor analysis and reliability analysis confirmed the unidimensionality of the scale. The IRT analysis showed the discriminative characteristics of the scale for those high and low in loneliness. The patterns of correlations of the scale with other variables provide ample evidence of the convergent validity of the scale.

Bimodality of the Test Information Curve (TIC) is the most important characteristic of the translated TIL Scale. The TIC has two distinct peaks for the latent trait (i.e. loneliness) over the desired range of the trait, implying that the short scale score is sufficiently informative to identify who the lonely or the non-lonely are. However, care should be exercised in interpreting the finding that Item 3 in the scale showed slight local dependence with the other two items, probably because of redundancy-dependency of the item contents (i.e., similar items are included in the same scale). Although the degree of overlap is not so large and should be interpreted in accordance with the number of items in the scale, this may produce overestimation of the scale parameter and underestimation of the location parameter of the item [25] and may result in overestimation of scale reliability [26]. Another point to mention is that the Item Information Curve (IIC) reveals that the item “I feel that I lack companionship” may not be effective for the discrimination of loneliness. The TIC and IICs of the items in the original English scale have not been reported yet, but future research is needed to analyze the characteristics of the original items based on large-scale responses obtained from English-speaking samples. The appropriateness of the Japanese language of the items should also be further examined.

The score of the translated TIL Scale was correlated with different indicators of loneliness, each of which theoretically explains the psychological concept from different perspectives. R-UCLA is the original of the TIL Scale, so the strong and positive correlation between R-UCLA and the translated TIL Scale is expected [10]. The small but significant associations of marital status and living arrangements with loneliness are consistent with the previous literature [20]. There was no gender difference in the loneliness score in the current samples.

In terms of age, the teens and twenties reported higher loneliness scores in the translated TIL Scale than older respondents in Sample 1, but not in Sample 2. A possible reason for the inconsistency of the findings between the samples is the difference in the proportion of youth to the whole sample. Sample 1 was drawn from an online research panel by using the equal allocation procedure for the age stratum, which could maximize the efficiency with which the mean and variance are estimated in each age group. Sample 2 was recruited through an online crowdsourcing service without controlling for demographic factors of the respondents, which may result in a relatively smaller number of teens and twenties than those in Sample 1. In addition, the respondents participated in the current surveys by registering themselves as an online research panel or crowdsourcing workers. This means that the older respondents in the current samples may be more active and familiar with communication technology, and thus less lonely [27], than ordinary samples of the same age drawn from the general population. On the other hand, the youth sample may be more lonely than ordinary samples in the same age group because they spent time on the internet to earn money, rather than socializing, by participating in the surveys. In sum, the age difference in loneliness found in Sample 1 may be a result of the comparison between the early adulthood group, who are generally high in loneliness due to their apprehensions about developmental tasks such as socialization, and old adults, who are relatively low in loneliness due to their skilled online activities.

The size of personal networks for social support was negatively related with the score of the translated TIL Scale, along with the total network size. This indicates a relationship between social isolation and loneliness [4]. Having an online network showed neither an incremental nor a decremental effect on loneliness, which could be the case when an overlap between offline and online personal networks is not considered [28, 29]. All the Big Five personality traits showed significant associations with loneliness, but, consistent with the previous literature [11], extraversion and neuroticism were the two main personality traits that explain the variance of loneliness. These findings suggest the sufficient validity of the scale.

Since this is an initial step in developing the short-form loneliness scale in Japan, there are still several limitations. First, the Japanese version of the TIL Scale was tested only among the online samples. Although the samples were large and diverse, additional research on a representative sample of the Japanese population is needed to examine the generalizability of the current factor structure and scale validity. Second, it is important to clarify if the loneliness chronicity measured by the scale is related to the functional impairment of psychobiological systems among the Japanese. There is strong relevance between the loneliness score obtained from the original TIL Scale and physiological functions, such as cardiovascular activation, cortisol levels, and immune responses [2]. Finding the link between subjective ratings and objective indicators of the negative feeling among the Japanese would give significant understandings of the roots of loneliness in a framework of cultural neuroscience [30].

## Conclusions

All in all, the above evidence demonstrates that the translated TIL Scale has sufficient characteristics for its factor structure and convergent validity to measure loneliness among Japanese people in a simplified format. Administration of the scale could be a plausible option, especially when respondents’ time and effort are major concerns of the research.

## Additional file

Additional file 1: **Table S1.** The Japanese version of the Three-Item Loneliness Scale. **Table S2.** Regression analysis of dummy variables (age) on the loneliness score (Sample 1). (DOCX 20 kb)

## Abbreviations

AIC: Akaike’s information criterion
GPCM: Generalized partial credited model
IIC: Item information curve
IRCCC: Item response category characteristics curve
IRT: Item response theory
PCM: Partial credited model
R-UCLA: The Revised UCLA (University of California, Los Angeles) Loneliness Scale
TIC: Test information curve
TIL Scale: The Three-Item Loneliness Scale

## Footnotes

1 Social network researchers distinguish the term “social networks” from “personal networks.” The former focuses on the “sociocentric” aspect of networks, referring to patterns of communications in a bounded group or organization as a whole, while the latter focuses on the “egocentric” aspect of networks, referring to the social ties each person holds [31].

2 GPCM is developed to analyze partially ordered categorical responses as an extension of 2-parameter logistic models. Although it is possible to apply the 1-parameter Partial Credit Model (PCM), in which the slope parameter is set as 1 across items, GPCM (AIC = 4973.7) showed a better fit to the data than PCM (AIC = 5045.0).
