# Supplementary material for: Development of the Japanese version of the three-item loneliness scale
Source: BMC Psychol. 2019 Apr 5;7:20. doi: 10.1186/s40359-019-0285-0 (PMC6449911; doi:10.1186/s40359-019-0285-0)
Supplement: Supplementary file 1 — Table S1. The Japanese version of the Three-Item Loneliness Scale. Table S2. Regression analysis of dummy variables (age) on the loneliness score (Sample 1). (DOCX 20 kb) [file 40359_2019_285_MOESM1_ESM.docx]

Supplementary Materials

Additional file 1: Table S1

The Japanese version of the Three-Item Loneliness Scale

Instruction

以下は、あなたがふだんの生活のさまざまな場面で、どのように感じているのかについての質問です。もっともあてはまる選択肢を回答してください。

Items

1. あなたは、自分に仲間付き合いがないと感じることがありますか

2. あなたは、疎外されていると感じることがありますか

3. あなたは、他の人から孤立していると感じることがありますか

Option

1: ほとんどない

2: たまにある

3: よくある

Additional file 1: Table S2

Regression analysis of dummy variables (age) on the loneliness score (Sample 1).

|  | *B* |  | 95%CI | *β* |  |
| --- | --- | --- | --- | --- | --- |
| (Intercept) | 5.74 | ** | [5.50, 5.97] | .000 |  |
| Demographic variables |  |  |  |  |  |
| Age (1 = twenties) | 0.00 |  | [-0.33, 0.33] | .001 |  |
| Age (1 = thirties) | -0.37 | * | [-0.70, -0.04] | -.085 |  |
| Age (1 = forties) | -0.47 | ** | [-0.80, -0.14] | -.109 |  |
| Age (1 = fifties) | -0.64 | ** | [-1.01, -0.26] | -.124 |  |
| Age (1 = sixties and older) | -0.99 | ** | [-1.45, -0.53] | -.146 |  |
| *R*^2^ |  |  |  | .031 | ** |
| *Note*. *N* = 1020. ***p* < .01, **p* < .05. The teens is the baseline of age. | | | | | |
